# Fetal Autopsy: Insights Into the Spectrum of Dysraphisms With Associated Anomalies

**DOI:** 10.7759/cureus.68147

**Published:** 2024-08-29

**Authors:** Kavitha K, Neelayadakshi Bhawani Shankar, Sudha Vasudevan

**Affiliations:** 1 Department of Pathology, Saveetha Medical College and Hospital, Saveetha Institute of Medical and Technical Sciences, Saveetha University, Chennai, IND

**Keywords:** single umbilical artery, anencephaly, fetal autopsy, congenital anomalies, neural tube defects

## Abstract

Introduction and aim

Malformations of the central nervous system (CNS) are one of the most devastating, yet most common congenital anomalies. Congenital CNS anomalies are structural or functional abnormalities of the brain and spinal cord that occur during the intrauterine developmental period and have a high intrauterine mortality. This study aimed to determine the frequency and distribution of neural tube defects (NTDs) in a tertiary care center for a period of 4.5 years. The neural tube defect was analyzed along with the other associated anomalies in the fetus. Its association with maternal risk factors was also analyzed.

Materials and methods

This retrospective study includes a total of 48 cases of neural tube defects, over a period of 4.5 years from January 2019 to June 2023, retrieved from the archives of the Department of Pathology at Saveetha Medical College. The study population included all fetuses diagnosed with NTDs during the fetal autopsies conducted at Saveetha Medical College during the period of study. The variables studied include maternal age, maternal risk factors, sex of the fetus, other associated anomalies in the fetus, and placental anomalies associated with the NTDs.

Results

A total of 48 cases of NTDs were included in this study. NTDs were more common among the female fetuses when compared with the male fetuses. The most common NTD observed in our study was anencephaly. The associated anomalies seen with NTDs include dysplastic kidney, unilateral renal agenesis, agenesis of urinary bladder, sacral agenesis, diaphragmatic hernia, bilateral talipes equinovarus deformity in both lower limbs. In the present study, among the placental defects seen in fetuses with NTDs, single umbilical artery was seen in four fetuses with NTDs. Twelve out of the 48 females included in the study did not take folic acid supplements during the antenatal period, and two females had a history of neural tube defects in their previous pregnancies.

Conclusion

This study emphasizes that intensive screening and documentation of coexistent abnormalities of NTDs is needed. Either ultrasonography/MRI or fetal autopsy should be conducted in order to exhibit certain diagnoses and to conduct proper prenatal genetic counseling for parents with fetuses having NTDs or having a history of fetuses with NTDs regarding ongoing and future pregnancies.

## Introduction

Malformations of the central nervous system (CNS) are one of the most devastating, yet most common congenital anomalies. Congenital anomalies of the central nervous system are structural or functional abnormalities seen in the brain and spinal cord, which occur during the intrauterine development of the fetus [1].

The incidence of malformations of the central nervous system is about one in 100 births [2]. Neural tube defects occur due to the failure of closure of the neural tube during the period of embryonic development between the third and fourth weeks of intrauterine development [1,3]. They have a high intrauterine mortality rate.

The purpose of this study was to determine the frequency and distribution of neural tube defects (NTDs) in a tertiary care center for a period of 4.5 years. The neural tube defects were analyzed along with the other associated anomalies that were present in the fetus and in the placenta. The association of neural tube defects with maternal risk factors was also analyzed.

## Materials and methods

This retrospective study was conducted in the Department of Pathology at Saveetha Medical College. It includes all fetuses with neural tube defects (NTDs) detected and confirmed during fetal autopsy, over a period of 4.5 years from January 2019 to June 2023. All fetal autopsies performed within the specified study period were analyzed, and the cases with neural tube defects were identified based on clinical history, prenatal imaging, and/or based on autopsy. Incomplete autopsy reports and cases with severe autolysis which hinder proper assessment of the neural tube defects were excluded from the study. A total of 48 cases with neural tube defects which were confirmed during fetal autopsy were identified and included in the study. The other required data was collected from the archived autopsy records, clinical case files, and histopathology slides. The variables collected include demographic details like gestational age and sex of the fetus, maternal age, gravida and parity, clinical details like any previous history of neural tube defects, any family history of congenital anomalies, and any relevant environmental exposures.

Standard fetal autopsy procedures were followed, with particular attention to the central nervous system. Detailed macroscopic examination findings, focusing on the presence or absence of neural tube defects, and the type of neural tube defects present, such as anencephaly, spina bifida, and encephalocele, were all noted. Additionally, the presence of any associated defects in these fetuses diagnosed with neural tube defects and any placental defects in these fetuses was also noted.

Data were entered into an Excel spreadsheet (Redmond, WA: Microsoft Corp.). Descriptive statistics was used to summarize the data. Frequencies and percentages were used to express categorical variables. The standard deviation, mean, median, and mode were used to represent continuous variables. The prevalence of different types of NTDs in the study population was calculated. Maternal and fetal factors associated with NTDs were also explored.

## Results

**Figure 1:**
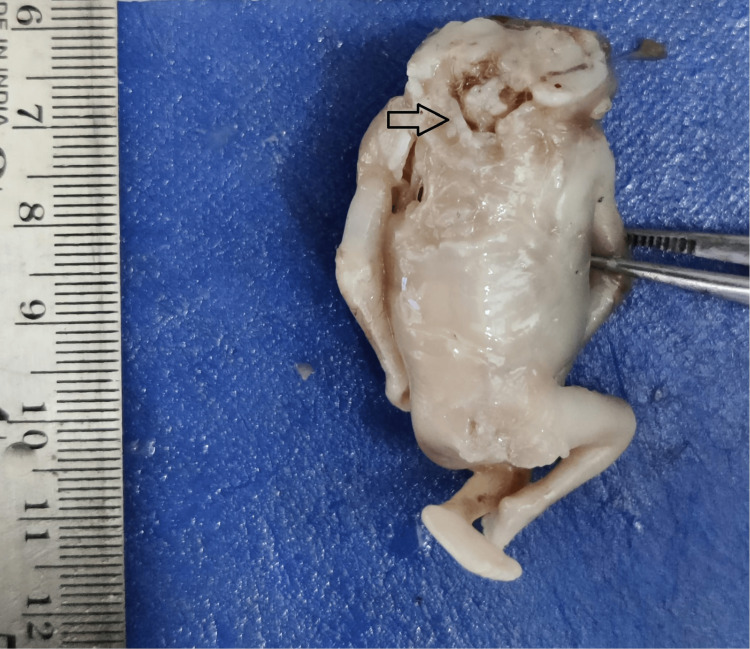
Fetus with anencephaly.

A total of 48 fetuses diagnosed with neural tube defects were included in this study. Neural tube defects were more common among female fetuses when compared with male fetuses. The mean age of the mother with a fetus having a neural tube defect in the present study was 27.3±3.6 years. The most common neural tube defect observed in our study was anencephaly (Figure 1).

**Figure 2:**
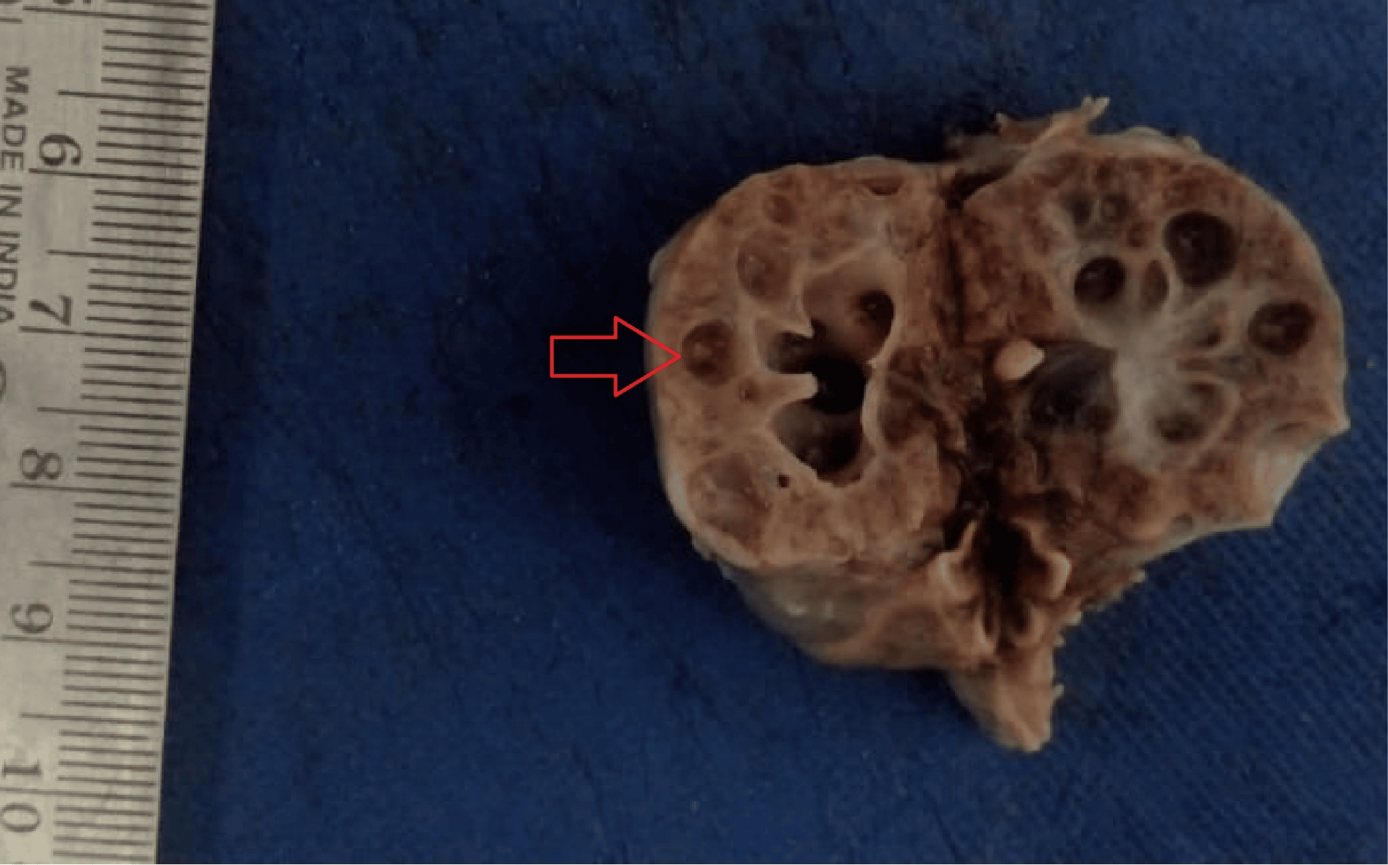
Cystic changes in the kidney. Cut surface of the kidney from a fetus with neural tube defect, showing multiple cysts (arrow).

**Figure 3:**
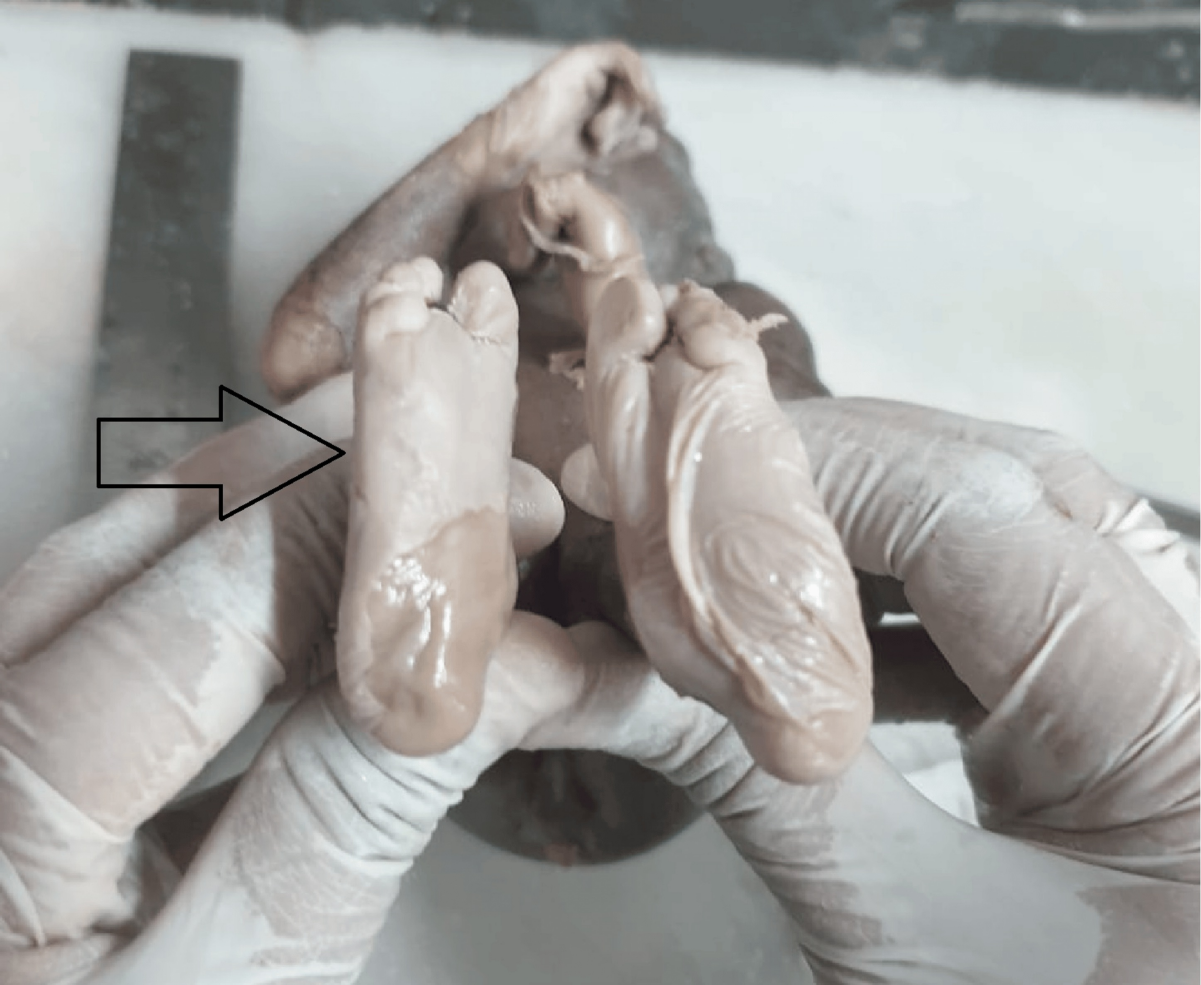
Bilateral talipes equinovarus deformity in both lower limbs with neural tube defect.

The associated anomalies seen with neural tube defects include multiple cysts in the kidney (Figure 2), dysplastic kidney, unilateral renal agenesis, agenesis of the urinary bladder, sacral agenesis, diaphragmatic hernia, bilateral talipes equinovarus deformity in both lower limbs (Figure 3).

Among the placental defects seen in fetuses with neural tube defects, a single umbilical artery was seen in four fetuses with neural tube defects in the present study. Anencephaly was most commonly associated with urinary tract abnormalities in the present study. The spectrum of neural tube defects observed in this study is shown in Table 1. 

**Table 1:** Spectrum of neural tube defects observed in the present study.

S. no.	Spectrum of neural tube defects	Number of cases	Percentage
1.	Anencephaly	18	37.5%
2.	Spina bifida/myelomeningocele	14	29.16%
3.	Arnold-Chiari malformation	5	10.41%
4.	Craniorachischisis	4	8.33%
5.	Encephalocele	4	8.33%
6.	Iniencephaly	1	2.08%
7.	Prosencephaly	1	2.08%
8.	Exencephaly	1	2.08%

Among the maternal risk factors, 12 out of the 48 females included in the study did not take folic acid supplements during the antenatal period and two females had a history of neural tube defects in previous pregnancies.

## Discussion

Development of the central nervous system in the fetus occurs during the first trimester, and the structures evolve over the next two trimesters [4]. Neural tube defects and other central nervous system malformations are the most common group of malformations that are detected in the prenatal period, and they account for a major proportion of all congenital anomalies [4,5]. Perinatal and fetal autopsies are helpful in identifying the exact cause of death and thereby recognizing those disorders that may require counseling for the management and planning of future pregnancies. Globally, around 303,000 newborn deaths occur within the first four weeks of birth every year due to congenital anomalies. The prevalence of abnormalities of the central nervous system is about 5-10/1,000 births. Seventy-five of the fetal deaths occur as a result of structural abnormalities in the central nervous system [5]. Neural tube defects are the second most common congenital disability after congenital heart defects [6]. The true incidence of neural tube defects is difficult to determine since most of the severe forms occur very early in embryonic development and they lead to spontaneous abortion [7].

**Figure 4:**
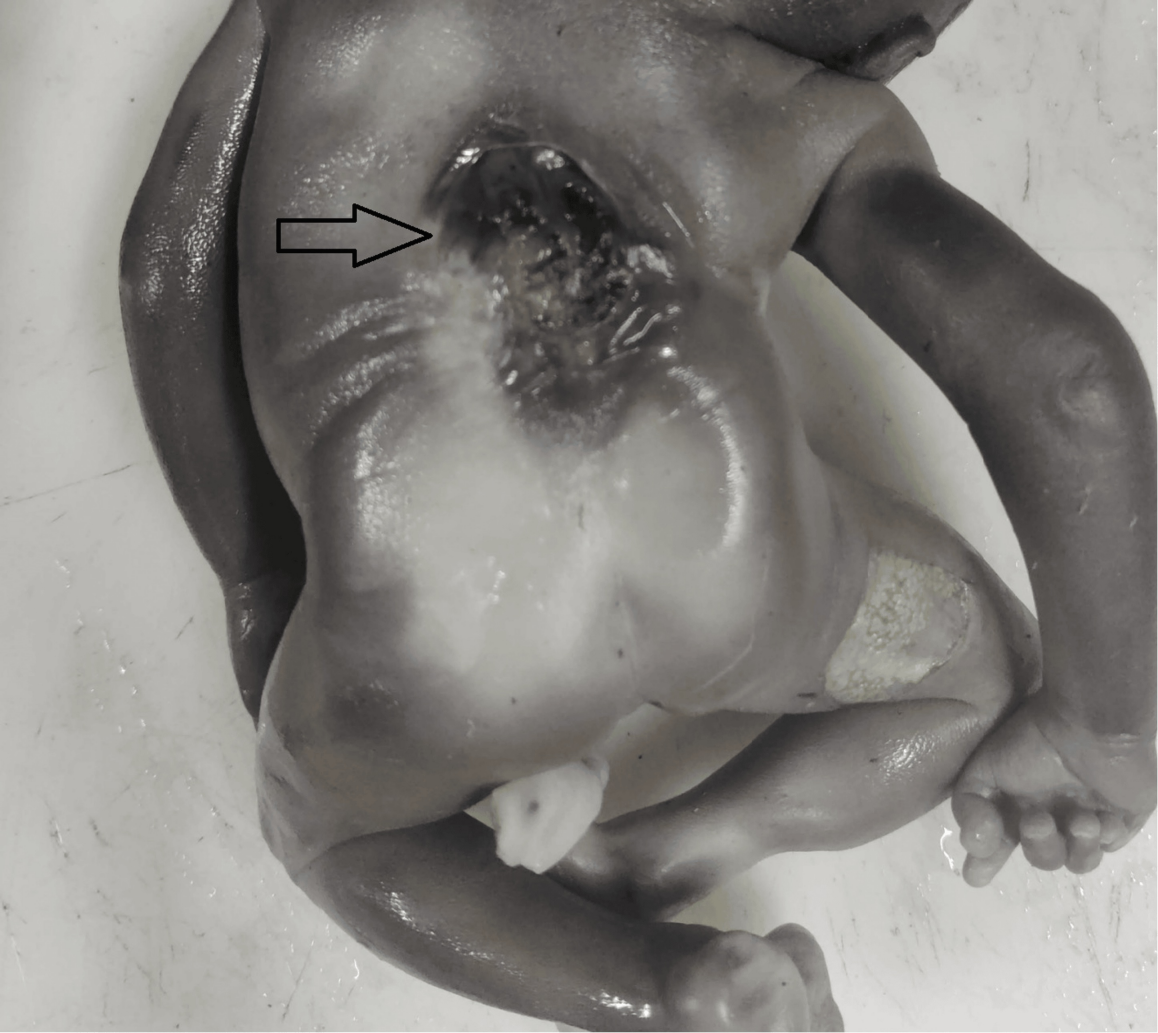
Spina bifida seen in the back of the fetus in the thoracic spine.

There are the following two types of NTDs: open and closed NTDs. The two most prevalent open NTDs are myelomeningocele, also known as open spina bifida, and anencephaly, with the latter being more common in the present study (Figure 4). Open neural tube defects (NTDs) in the spine may be associated with cerebral ventriculomegaly. Closed NTDs (defect is covered by skin) include lipomyelomeningocele and lipomeningocele. In Closed NTDs, the defect is covered with skin, although they can also have a tuft of hair, dimple, birthmark, lump, or other skin anomaly at the defect site. They can also be associated with cerebral ventriculomegaly and Chiari malformations as well [8].

**Figure 5:**
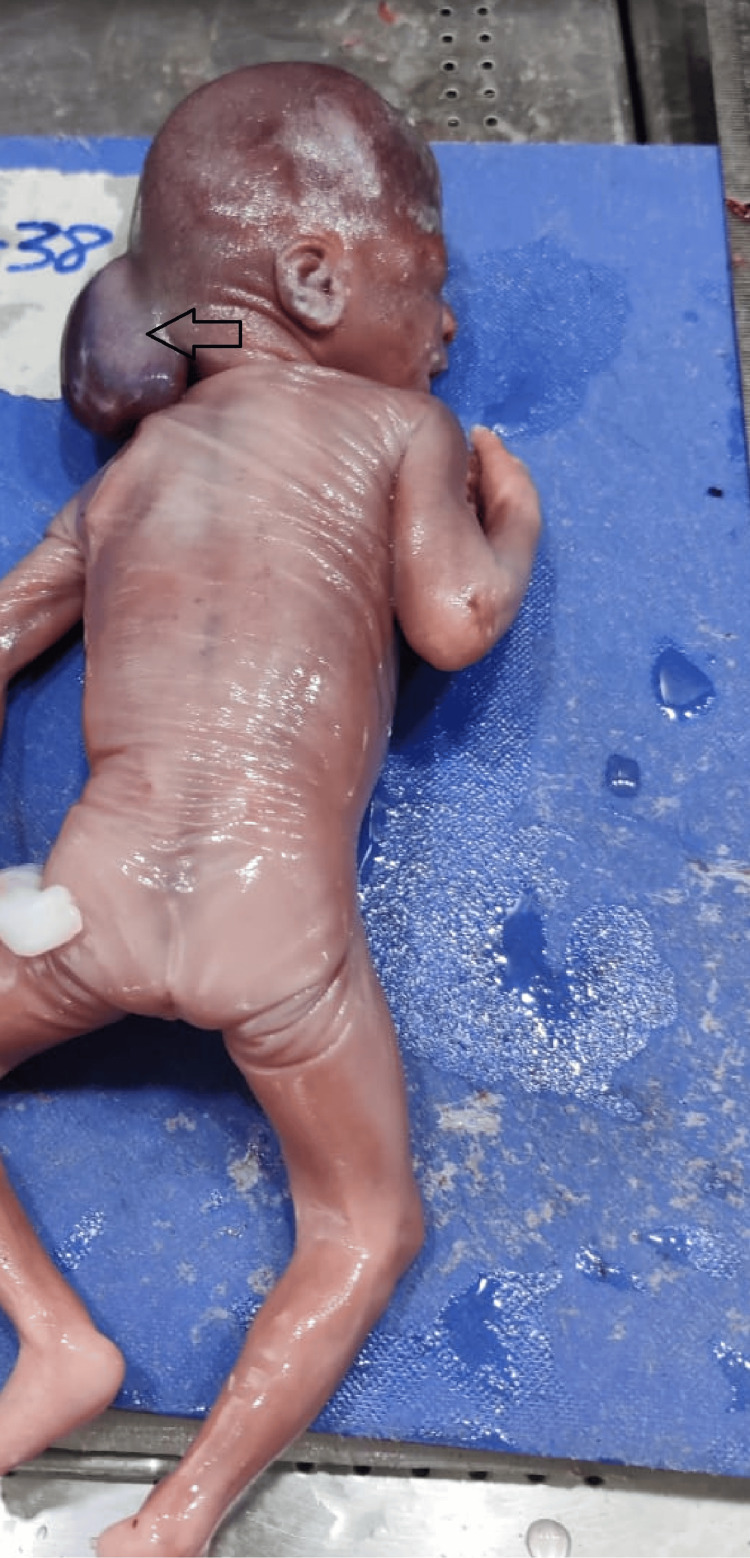
Encephalocele seen in the occipital region of the fetus.

Based on the location, NTD can be cranial defects or spinal dysraphism. Cranial defects include anencephaly, exencephaly, and encephalocele. When the cephalic end of the neural tube fails to close, it results in anencephaly where there is an absence of the forebrain, mainly the cerebral hemisphere along with the neocortex [9]. Exencephaly is the presence of an exposed neural tissue mass and encephalocele is the herniation of the intracranial contents through a defect present in the dura and the skull [10]. The most common form of encephalocele is occipital encephalocele, also seen in the present study (Figure 5) [11].

The exencephaly-anencephaly sequence is multi-factorial. First, there is a failure of closure of the anterior neural groove around 10-20 days post-ovulation, which is known as dysgraphia. Following this, as the development continues, a relatively normal-appearing brain is seen, which lacks a covering calvarium and meninges. This is known as exencephaly. Following this, the brain which is exposed to the amniotic fluid, disintegrates due to the chemical and mechanical effects of the amniotic fluid on the fetal brain. This is associated with the failure of development of the cerebral hemispheres and the skull, leading to the formation of anencephaly [12].

Congenital fissure involving the skull and vertebral column is known as craniorachischisis [13]. It is a very severe form of neural tube defect, characterized by the presence of anencephaly along with spina bifida, which is open from the cervical vertebral region to the lumbar or sacral vertebral region [14].

Spinal cord dysraphism refers to aberrations in the embryogenesis of the spine and spinal cord. Open spinal dysraphism (spina bifida aperta) is characterized by a cleft in the spinal column with herniation of the meninges (meningocele) or meninges and spinal cord (myelomeningocele) through the defect. Closed spinal dysraphism is also known as occult spinal dysraphism or spina bifida occulta. Open or closed spinal dysraphism occurs at a frequency of 0.5-8 cases per 1,000 live births [15]. Arnold-Chiari malformations are abnormalities of the posterior fossa that involve the region of the brain where the brain and spinal cord meet. It affects the cerebellum, brain stem, and the spinal cord. Arnold-Chiari malformation has a prevalence of 0.1-0.5% [16].

**Figure 6:**
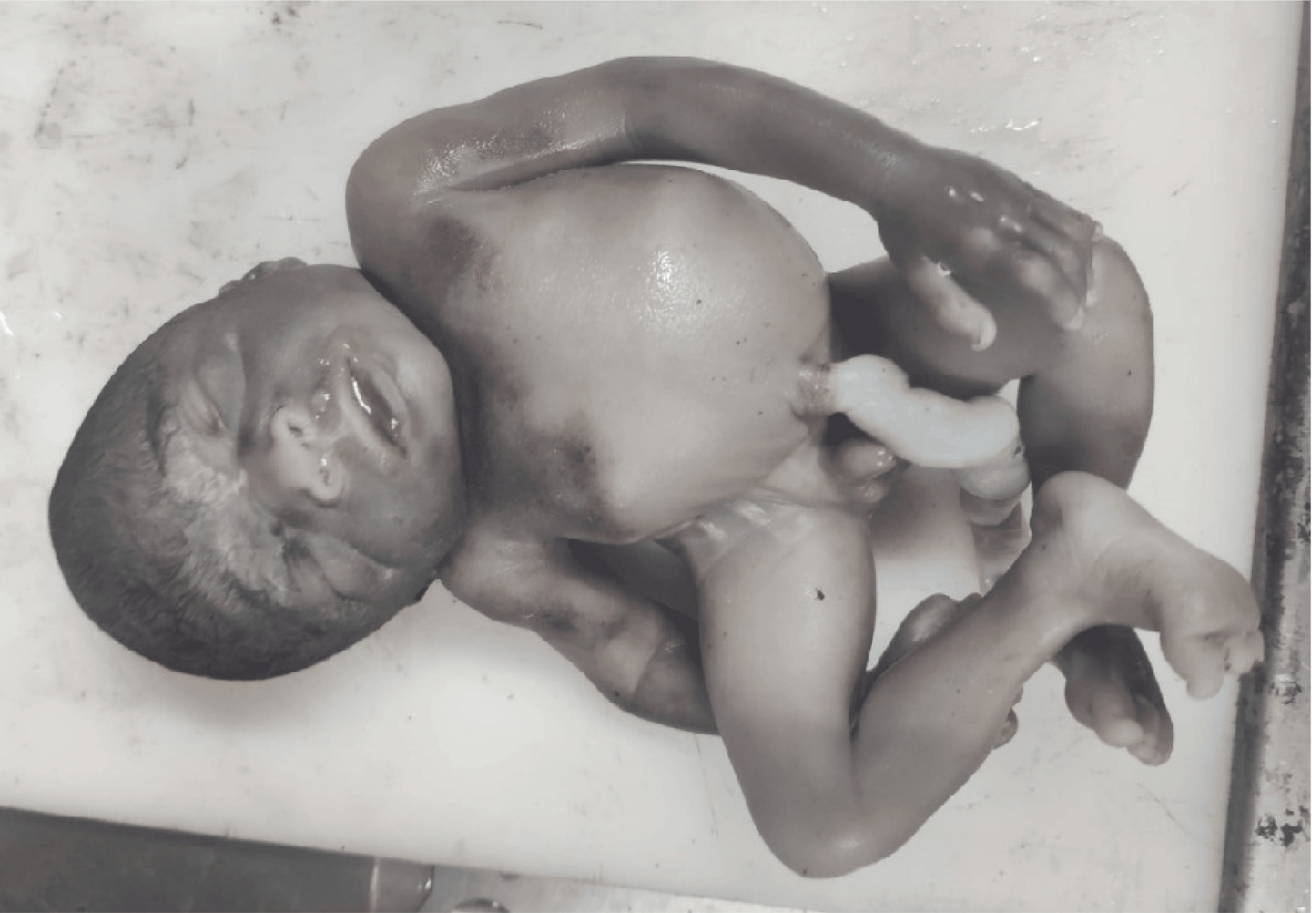
The fetus diagnosed with iniencephaly had retroflexed head with short neck and lordosis of the cervical vertebra.

Iniencephaly refers to a severe congenital anomaly characterized by abnormality of the craniovertebral junction. In these fetuses the head is retroflexed. The changes involving the skull and vertebra in iniencephaly include marked lordosis involving the cervical vertebrae, an irregular fusion of cervical vertebrae, duplication of cervical vertebra, small posterior fossa, and widening of the foramen magnum. The fetus with iniencephaly appears to have no neck since the skin in the back and the posterior scalp are continuous with each other [17]. In the fetus diagnosed with iniencephaly in our study, the fetus had retroflexed head with a short neck, lordosis of the cervical vertebra, along with a defect in the mid-to-lower back measuring, 3.5 x 2.2 cm, and sacral agenesis (Figure 6).

**Figure 7:**
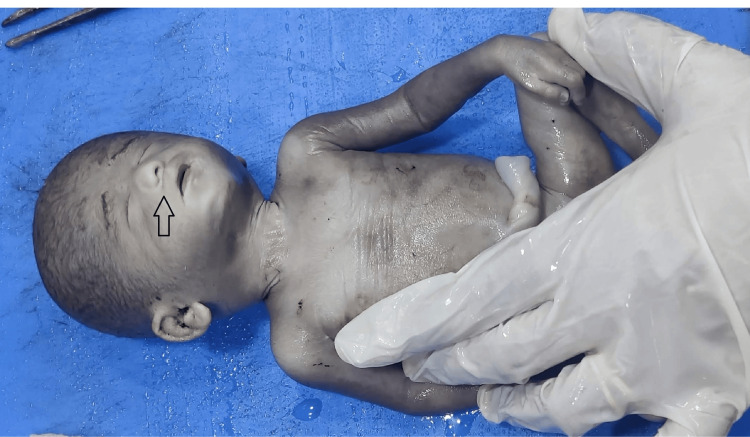
Prosencephaly - the fetus had a single nostril along with hypotelorism and a flat nose.

**Figure 8:**
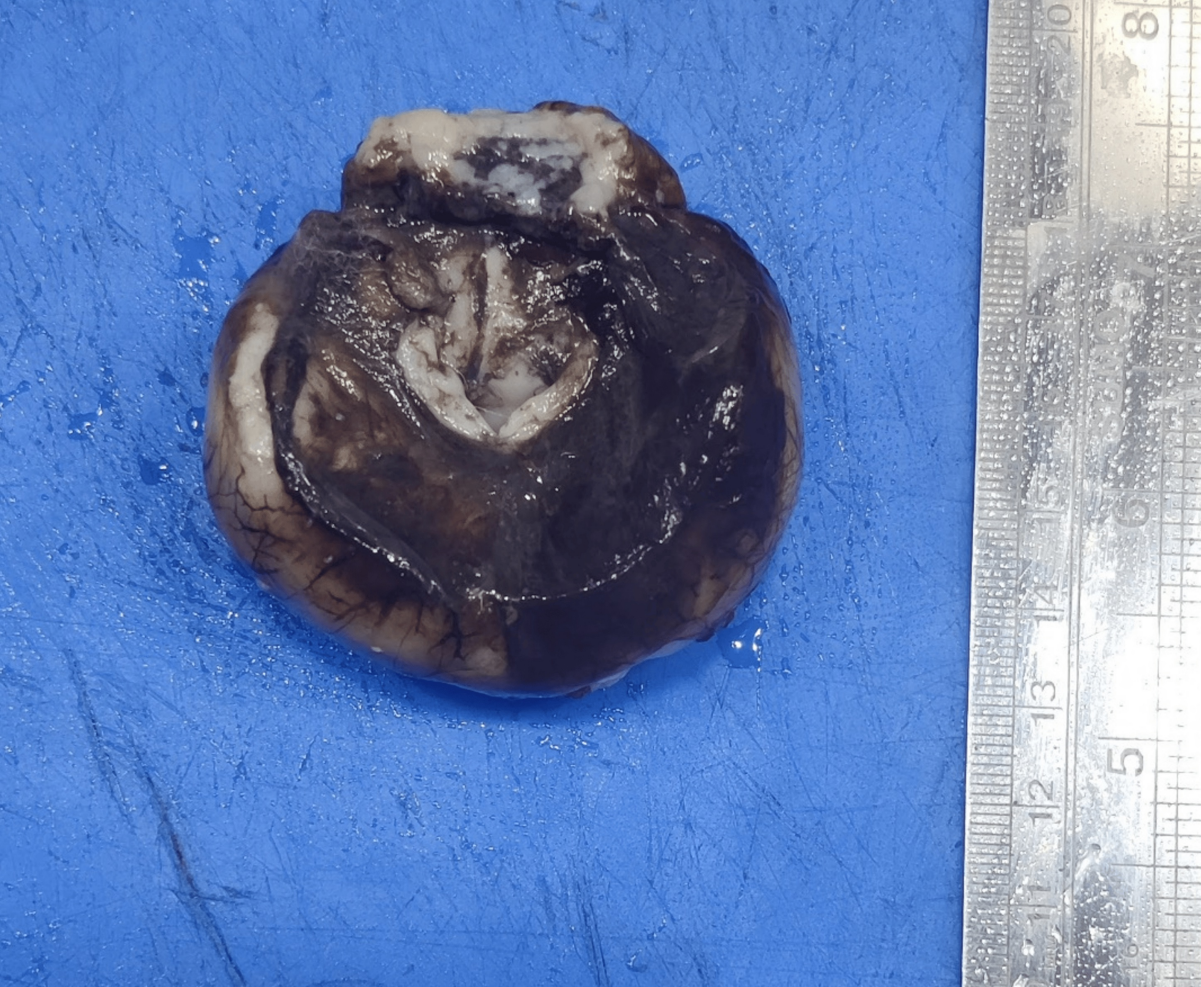
An alobar brain from a fetus with prosencephaly. Gross specimen of the brain from a fetus diagnosed with prosencephaly showing alobar brain with no separation of the cerebral hemispheres.

Holoprosencephaly is a CNS anomaly caused by impaired mid-line cleavage of the forebrain in the fetus. It is usually associated with facial anomalies in the fetus, with cyclopia being an extreme variant [18]. The fetus diagnosed with prosencephaly in our study was corresponding to 22 weeks gestation. The fetus had a single nostril (Figure 7). The fetus was diagnosed with hypotelorism, flat nose, and meningocele (spina bifida occulta was present) in the sacral region. The brain was alobar, no sulci were seen, and vermis was present (Figure 8).

The most common CNS anomaly seen in the present study is anencephaly, which is similar to the studies done by Nielsen et al. and Kar et al. [3,9]. In the study done by Toru et al., the most common central nervous system anomaly seen was craniorachischisis [19]. However, contrary to our study, the most common central nervous system anomaly seen in the studies done by Vinutha et al. and Pinar et al. was meningomyelocele [1,2]. Also in the study done by Siddesh et al., the most common central nervous system anomaly noted was spina bifida [4].

The mean age of the mother, whose fetus was diagnosed with neural tube defects in our study was found to be 27.3 years, which was almost similar to the observations noted in other studies, such as Vinutha et al. (26-30 years), Siddesh et al. (28 years), Toru et al. (26.6 years), and Kar et al. (25-29 years) [1,4,19,9].

The most commonly affected sex of the fetus with neural tube defects was found to be the female sex in the present study. Similarly, the most common sex of the fetus affected with neural tube defect in the studies done by Toru et al. and Kar et al. was the female sex [19,9]. On the contrary, the most common sex of the fetus affected with neural tube defect in the studies done by Vinutha et al., Nielsen et al., and Pinar et al. was the male sex (Table 2) [1-3].

**Table 2:** Comparison of variables studied in the the various studies on NTD. NTD: neural tube defect

Variables	Present study	Vinutha et al. [1]	Siddesh et al. [4]	Nielsen et al. [3]	Toru et al. [19]	Kar et al. [9]	Pinar et al. [2]
Most common NTD seen	Anencephaly	Meningomyelocele	Spina bifida	Anencephaly	Craniorachischisis	Anencephaly	Meningocele/meningomyelocele
Mean age of the mother	27.3 years	26-30 years	28 years	-	26.6 years	25-29 years	-
Most commonly affected sex in the fetus with NTD	Female	Male	-	Male	Female	Female	Male
Sample size of fetuses with NTD	48	17	243	183	62	72	363

The limitations of the present study are the lack of chromosomal analysis and the small number of cases included in the study. Chromosomal analysis was not possible in these cases due to the financial constraints, and the non-consent of the parents for spending for further investigations for the babies, which have not survived. Additionally, transporting the samples to other institutions for karyotyping without causing disintegration was challenging.

## Conclusions

This study focuses on the importance of fetal autopsy along with ultrasound study for the primary prevention of NTDs. It emphasizes that intensive screening and documentation of coexistent abnormalities of NTDs is needed. Either ultrasonography, MRI, or fetal autopsy should be conducted to establish certain diagnoses and to provide proper prenatal genetic counseling for parents of fetuses with NTDs, or those with a history of NTDs, regarding the ongoing pregnancy and the management of future pregnancies. Fetal and perinatal autopsy can be very helpful in finding the cause of death and also serves as an audit for those anomalies, which could not be picked up by antenatal ultrasound.
